# The basic helix-loop-helix transcription factor TabHLH1 increases chlorogenic acid and luteolin biosynthesis in *Taraxacum antungense* Kitag

**DOI:** 10.1038/s41438-021-00630-y

**Published:** 2021-09-01

**Authors:** Qun Liu, Li Li, Haitao Cheng, Lixiang Yao, Jie Wu, Hui Huang, Wei Ning, Guoyin Kai

**Affiliations:** 1 Laboratory for Core Technology of TCM Quality Improvement and Transformation, College of Pharmacy, School of Pharmaceutical Sciences, The Third Affiliated Hospital, Zhejiang Chinese Medical University, Hangzhou, Zhejiang 310053 PR China; 2 College of Horticulture, Shenyang Agricultural University, Shenyang, 110866 China; 3 Institute of Botany, Jiangsu Province and Chinese Academy of Sciences (Nanjing Botanical Garden Mem.Sun Yat-Sen), Nanjing, 210014 China; 4 College of Traditional Chinese Materia Medica, Shenyang Pharmaceutical University, Shenyang, 110016 PR China; 5 Guangxi Botanical Garden of Medicinal Plants, Nanning, 530023 PR China; 6 College of Life Sciences and Engineering, Shenyang University, Shenyang, 110044 PR China

**Keywords:** Metabolic engineering, Secondary metabolism

## Abstract

Polyphenols are the main active components of the anti-inflammatory compounds in dandelion, and chlorogenic acid (CGA) is one of the primary polyphenols. However, the molecular mechanism underlying the transcriptional regulation of CGA biosynthesis remains unclear. Hydroxycinnamoyl-CoA:quinate hydroxycinnamoyl transferase *(*HQT2) is the last rate-limiting enzyme in chlorogenic acid biosynthesis in *Taraxacum antungense*. Therefore, using the *TaHQT2* gene promoter as a probe, a yeast one-hybrid library was performed, and a basic helix-loop-helix (bHLH) transcription factor, *TabHLH1*, was identified that shared substantial homology with *Gynura bicolor* DC *bHLH1*. The *TabHLH1* transcript was highly induced by salt stress, and the TabHLH1 protein was localized in the nucleus. CGA and luteolin concentrations in *TabHLH1*-overexpression transgenic lines were significantly higher than those in the wild type, while CGA and luteolin concentrations in TabHLH1-RNA interference (RNAi) transgenic lines were significantly lower. Quantitative real-time polymerase chain reaction demonstrated that overexpression and RNAi of *TabHLH1* in *T. antungense* significantly affected CGA and luteolin concentrations by upregulating or downregulating CGA and luteolin biosynthesis pathway genes, especially *TaHQT2*, 4-coumarate-CoA ligase (*Ta4CL*), chalcone isomerase (*TaCHI*), and flavonoid-3′-hydroxylase (*TaF3*′*H*). Dual-luciferase, yeast one-hybrid, and electrophoretic mobility shift assays indicated that TabHLH1 directly bound to the bHLH-binding motifs of *proTaHQT2* and *proTa4CL*. This study suggests that TabHLH1 participates in the regulatory network of CGA and luteolin biosynthesis in *T. antungense* and might be useful for metabolic engineering to promote plant polyphenol biosynthesis.

## Introduction

Dandelions (*Taraxacum* spp.) have been used as medicinal herbs and functional foods for several centuries^1,2^. The increasing demand for dandelion products, such as tea, wine, syrup, and coffee, has promoted the industrialization of dandelion production^3^. The total phenolic compound concentrations in different tissues of *Taraxacum mongolicum* ranged from 37.12 to 68.89 mg GAE/g^4–7^. However, these levels in *Taraxacum antungense* have not been tested in previous studies. Polyphenolic compounds, including phenolic acids, flavonoids, and anthocyanins, have many biological activities^8–10^. In *Taraxacum antungense* Kitag, chlorogenic acid (CGA) and caffeic acid (CA) have antioxidative benefits, being hepatoprotective and having diuretic activities^11–13^; rutin and luteolin are used to treat several diseases, such as Parkinson’s disease, severe acute respiratory syndrome, hepatitis, and cancer^14–16^. However, the concentration of these functional active constituents in *T. antungense* is lower than that in other medicinal plants, such as *Lonicera japonica* and *Eucommia ulmoides*, which restricts dandelion industrialization^14^. Bioengineering strategies could potentially increase the polyphenolic compounds in *Taraxacum*; however, a better understanding of the polyphenolic compound biosynthesis pathway is required.

**Fig. 1:**
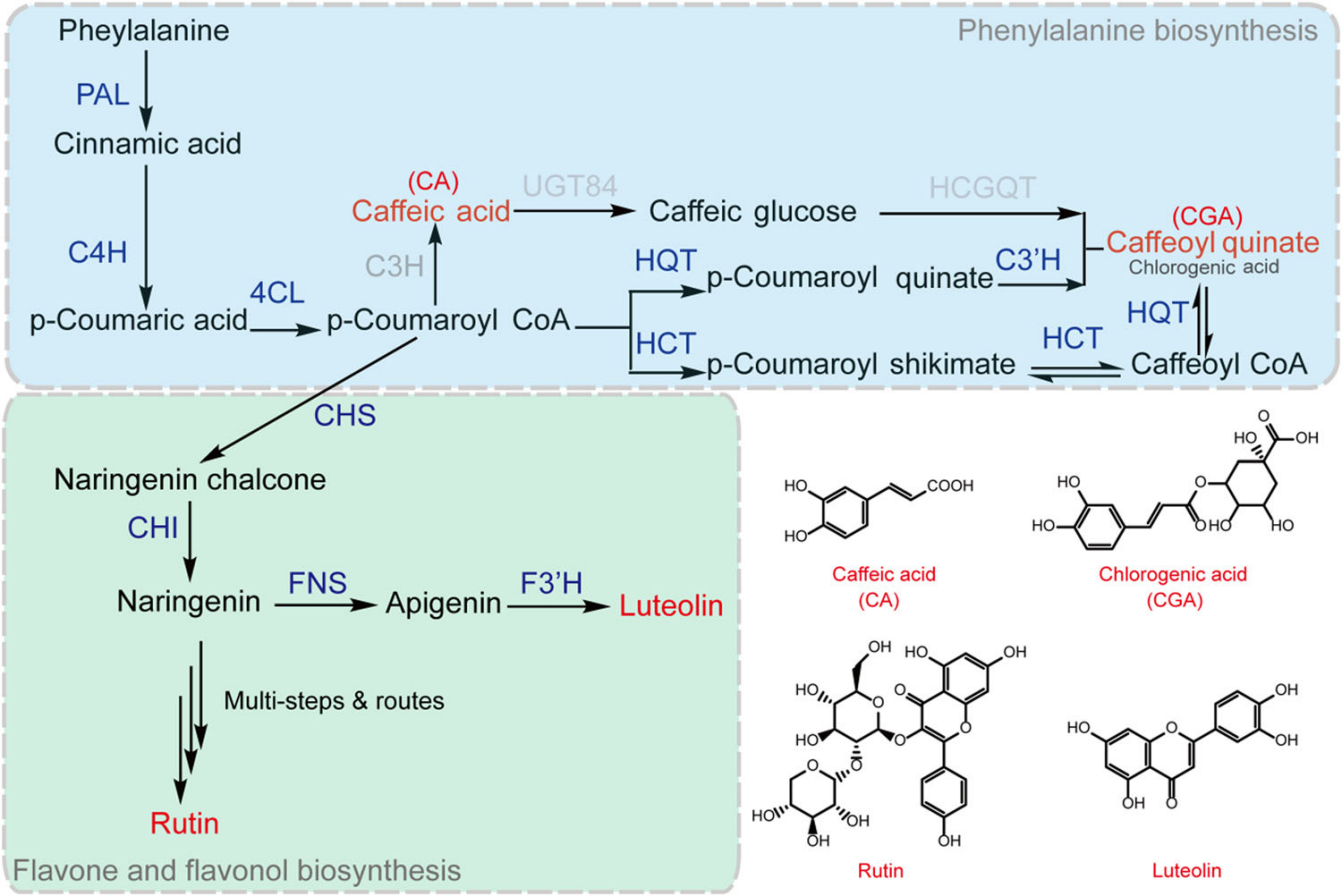
Biosynthetic pathway of the main polyphenols (caffeic acid, chlorogenic acid, rutin, and luteolin) in *Taraxacum antungense*. Phenylalanine ammonia-lyase (PAL); cinnamate 4-hydroxylase (C4H); 4-coumarate-CoA ligase (4CL); Coumarate 3-hydroxylase (C3H); 5-O-(4-coumaroyl)-D-quinate 3′-monooxygenase (C3′H); hydroxycinnamoyl-coenzyme (Co)A shikimate/quinate hydroxycinnamoyl transferase (HCT); hydroxycinnamoyl-CoA quinate hydroxycinnamoyl transferase (HQT); caffeoylshikimate esterase (CSE); UDP-glucosyltransferase (UGT84); Chalcone synthase (CHS); flavonoid synthase (FNS); Chalcone isomerase (CHI); and hydroxycinnamoyl D-glucose quinate hydroxycinnamoyl transferase (HCGQT). Three arrows indicate multi-steps & routes. Blue indicates what has been reported in this article, and gray indicates information from other plants

Since the 1990s, the polyphenol biosynthesis pathway has been reported in several medicinal model plants, such as *Salvia miltiorrhiza*, *L. japonica*, and *Dendranthema morifolium*^17,18^. In the first three steps of polyphenol biosynthesis, the key enzymes are phenylalanine ammonia-lyase (PAL), 4-coumarate-CoA ligase (4CL), and cinnamate 4-hydroxylase (C4H), which catalyze the synthesis of p-coumaroyl-CoA from phenylalanine^19^. CA is directly catalytically synthesized by coumarate 3-hydroxylase from p-coumaroyl-CoA, whereas CGA is catalytically synthesized by hydroxycinnamoyl CoA quinate hydroxycinnamoyl transferase (HQT) and hydroxycinnamoyl-CoA shiki-mate/quinate hydroxycinnamoyl transferase (HCT)^20^. However, in *T. antungense*, only HQTs (*TaHQT1*/*2*) have been isolated, identified, and assessed, and TaHQT2 is the last rate-limiting enzyme in chlorogenic acid biosynthesis^21^. For flavonoids, p-coumaroyl-CoA is a branch and precursor compound. The first two steps in the catalytic synthesis of naringenin from p-coumaroyl-CoA are via chalcone synthase and chalcone isomerase (CHS and CHI, respectively)^8^. Naringenin is catalyzed by flavonoid synthase and flavonoid-3′-hydroxylase (FNS and F3′H, respectively) in plants to synthesize luteolin^8,22^. Naringenin is also catalyzed by flavonol-3-dehydrogenase (F3H), flavonol synthetase (FLS), and UDP-glucoronosyl/UDP-glucosyl transferase (UFGT) to synthesize rutin^23–25^. PAL, CHS, CHI, and F3′H increase CGA and luteolin concentrations in *L. japonica*, whereas in *Fagopyrum tataricum*, the rutin concentration is increased by upregulation of FLS and UFGT gene expression^25,26^. Identification of these polyphenol biosynthesis pathway genes will lay the foundation for further genetic engineering research in *T. antungense* (Fig. 1).

Plant polyphenolic compounds are important components acting against biotic and abiotic stresses^21,25^. Plant transcription factor (TF) family members, such as MYB11/12/111 and WRKY18/40/60, respond to biotic and abiotic stress to regulate the expression of downstream structural genes and ultimately promote the biosynthesis of polyphenols^18,19,27^. Coexpression analysis of TFs and biosynthesis pathway gene expression levels often showed a highly linear relationship^27,28^. Thus, TFs may be used as a tool not only to improve a plant’s ability to adapt to the environment but also to increase polyphenolic compound production in plants^21^.

Basic helix-loop-helix (bHLH) TFs are one of the largest families that regulate the expression of key enzyme genes in plants^28^. Among these TFs, myelocytomatosis oncogene (MYC) TFs (representative bHLH TFs) often participate in secondary metabolite accumulation: AaMYC2 (*Artemisia annua* L) regulates artemisinin biosynthesis, NtMYC2 (*Nicotiana tabacum*) regulates nicotine biosynthesis, and CrMYC2 (*Catharanthus roseus*) regulates the expression of alkaloid biosynthesis genes that respond to methyl jasmonate (MeJA)^29,30^. In the medicinal model plant *S. miltiorrhiza*, *SmMYC2a*/*2b* regulates key enzyme genes for phenolic acid biosynthesis^31^. Upregulation of *SmPAL* by *SmbHLH37* leads to increased phenolic acid accumulation^32^. Moreover, the MeJA-responsive *SmbHLH53* TF regulates enzymatic genes involved in the salvianolic acid B biosynthesis pathway in *S. miltiorrhiza*^33^. However, the function of bHLH TFs in *T. antungense* polyphenol biosynthesis and their regulatory models have been poorly reported^34^.

TFs regulate gene expression levels by combining with cis-acting elements of functional gene promoters; bHLH TFs specifically bind to E-box^28,35^. In *T. antungense*, the *TaHQT2* gene promoter was obtained and was found to contain various cis-acting elements, specifically four E-boxes (CANNTG) (Supplementary Fig. S1 and Table S2). E-boxes are widely distributed in the promoter region of key enzymes in polyphenol biosynthesis^36^. Therefore, it was speculated that bHLH TFs bind to the *TaHQT2* promoter to participate in polyphenol biosynthesis in *T. antungense*.

In this study, a *T. antungense* bHLH TF, *TabHLH1*, which was obtained through yeast one-hybrid (Y1H) screening, had high homology to *bHLH1* from the *Gerbera hybrid cultivar*. Polyphenol concentration analysis and quantitative real-time polymerase chain reaction (qRT-PCR) results showed that *TabHLH1* increased CGA and luteolin biosynthesis by increasing *TaHQT2*, *Ta4CL*, *TaCHI*, and *TaF3*′*H* gene expression levels in *T. antungense* transgenic lines. Functional analysis of *TabHLH1* suggested that it regulates the biosynthesis of CGA and luteolin, enhancing the understanding of the routes of polyphenol biosynthesis and providing a structure for future metabolic engineering of *T. antungense*.

## Results

### Isolation and characterization of TabHLH1

**Fig. 2:**
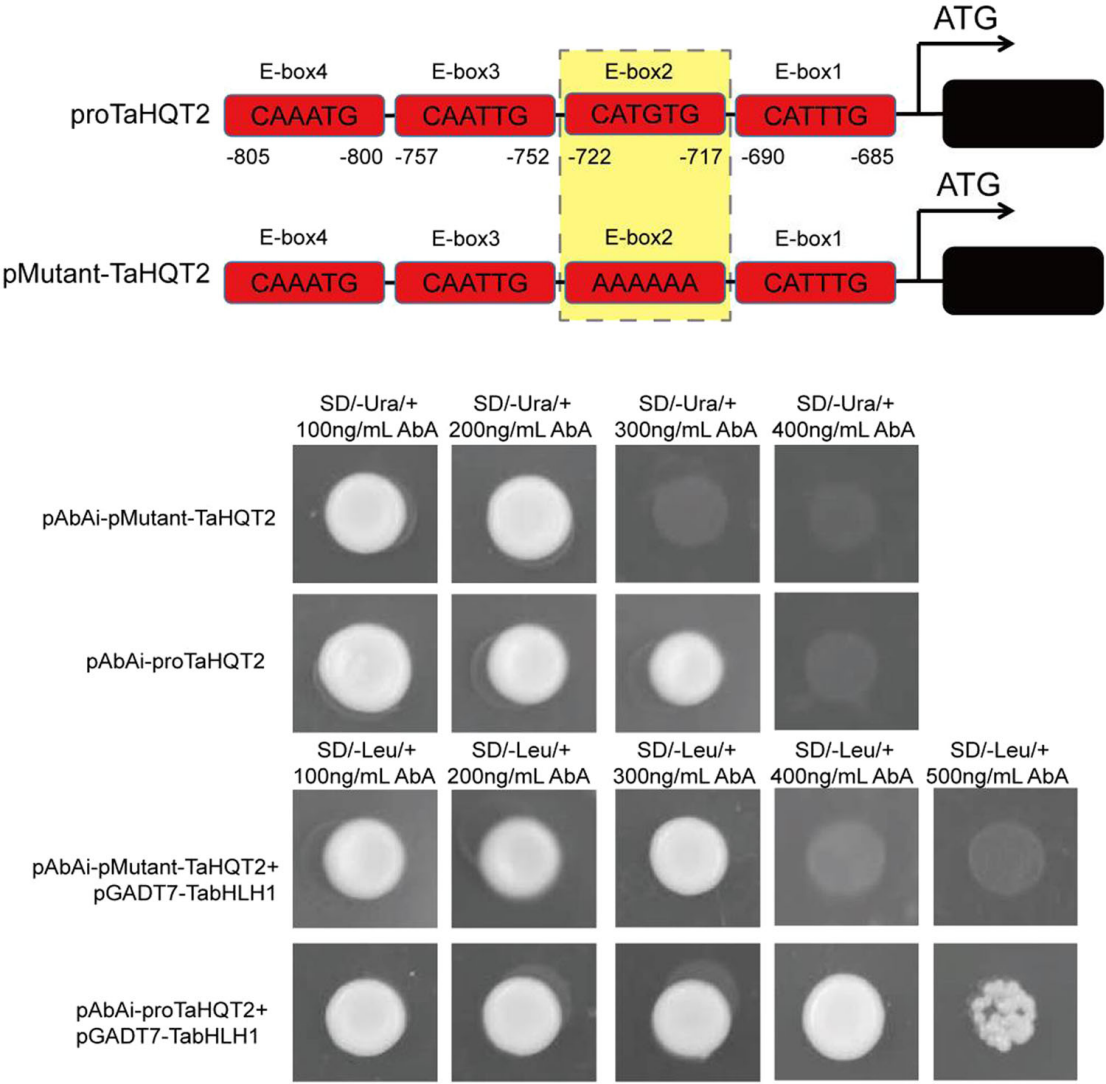
Y1H assay. pAbAi-E-box grew normally on 300 ng/mL aureobasidin A (AbA); however, pAbAi-pMutant-E-box was inhibited by 300 ng/mL AbA. Therefore, 400 ng/mL was the minimum concentration of AbA used to examine the interaction between TabHLH1 and the E-boxes. We used the pAbAi-E-box and pAbAi-pMutant-E-box to transform Y1H gold yeast and then made new Y1H competent cells. Subsequently, the pGADT7-TabHLH1 recombinant vector was transformed into new Y1H competent cells. The cotransformed pAbAi-E-box + pGADT7-TabHLH1 grew on 400–500 ng/mL AbA; however, pAbAi-pMutant-E-box + pGADT7-TabHLH1 was unable to grow and needed a higher concentration of AbA. The difference between the pAbAi-E-box and pAbAi-pMutant-E-box was that the E-box pAbAi-E-box contained CAAATG, CATTTG, CATGTG, and CAATTG motifs. In the pAbAi-pMutant-E-box, AAAAAA was used instead of the CATGTG motif

To identify bHLH TFs involved in CGA biosynthesis of *T. antungense*, Y1H assays were applied to screen the *T. antungense* cDNA library, and the *TaHQT2* promoter was used as bait. Approximately 860 bp of the *TaHQT2* promoter sequence (proTaHQT2) was cloned after two rounds of amplification, and several elements were identified, including an androgen response element, TATA-box, CAAT-box, CGTCA/TGACG-motif, estrogen-responsive element, long terminal repeat, E-box, P-box, TGA-box, and light-responsive elements (Fig. S1 and Table S2). A Y1H cDNA library of *T. antungense* was created with a titer of approximately 5 × 10^7^ colony-forming units/mL. PCR results showed that the length of *T. antungense* cDNA ranged from 200–2000 bp (Fig. S2). A 200 bp DNA fragment containing four E-boxes (proTaHQT2-E-box-1, -2, -3, and -4) that were identified in proTaHQT2 (from –685 to –810) within 860 bp of the ATG start codon was cloned. The isolated gene coding protein was able to bind to the proTaHQT2 CATGTG motif (Fig. 2). The results also showed that pMutant-TaHQT2 interacted with the isolated gene coding protein.

**Fig. 3:**
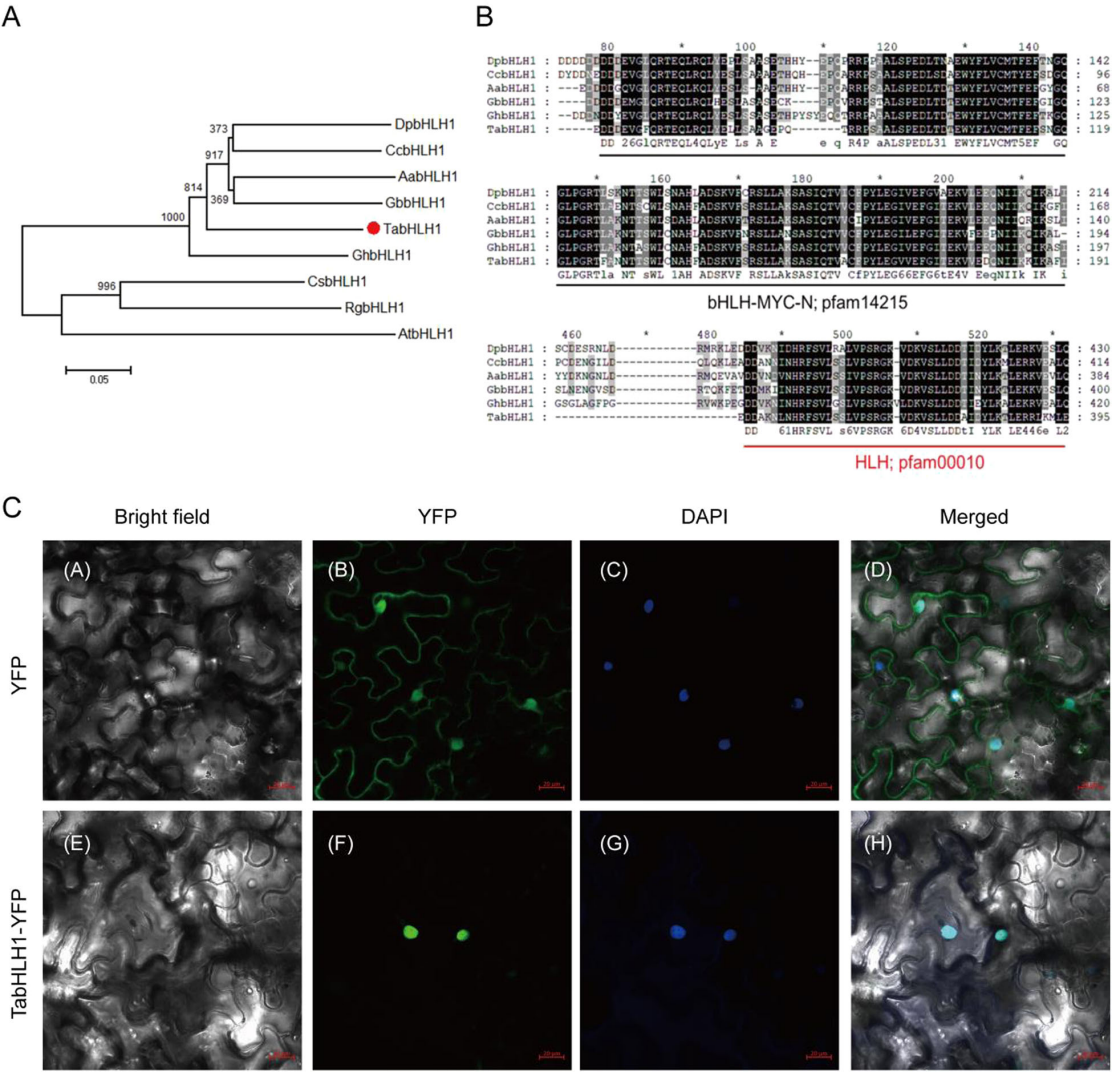
Characterization and subcellular localization of TabHLH1. **A** Phylogenetic analysis of TabHLH1 and related bHLH TFs. **B** Sequence alignment of TabHLH1 sequences in *T. antungense*. TabHLH1 (in this article, QBQ52949.1; *Taraxacum antungense* bHLH1), GhbHLH1 (CAA07615.1; *Gerbera hybrid* cultivar bHLH1), GbbHLH1 (BAJ17663.1; *Gynura bicolor* bHLH1), AabHLH1 (PWA53551.1; *Artemisia annua* bHLH1), DpbHLH (BAJ33516.1; *Dahlia pinnata* bHLH), CcbHLH1 (KVH95430.1; *Cynara cardunculus* var. scolymus bHLH1), CsbHLH1 (AOY10782.1; *Camellia sinensis* bHLH1), RgbHLH1 (ATZ76571.1; *Rubus genevieri* bHLH1) and AtbHLH1 (AAB72192.1; *Arabidopsis thaliana* bHLH1). The conserved domains pfam14215 (bHLH-MYC-N) and pfam00010 (HLH) are indicated by straight lines. The neighbor-joining method in MEGA software (version 8.0) was used to construct the phylogenetic tree. Bootstrap percentage (1000 replicates) values are listed above the branches. The TabHLH1 protein is labeled with a red circle. The black line indicates the bHLH-MYC-N motif; pfam14215. The red line indicates the HLH motif; pfam 00010. **C** Subcellular localization of the TabHLH1 protein in *N. benthamiana* leaves. Tobacco leaf cells were transiently transformed with recombinant plasmids containing TabHLH1-YFP or YFP (control group). DAPI staining indicates localization in the nucleus

The isolated protein acquired from the nonredundant protein database (using the Basic Local Alignment Search Tool for protein [BLASTP]) showed high homology to the *Gerbera hybrid cultivar bHLH1* (*GhbHLH1*: 69% identity and 78% positivity) and *Gynura bicolor bHLH1* (*GbbHLH*: 66% identity and 76% positivity) (Fig. 3A). The full-length *TabHLH1* cDNA was 1566 bp and encoded a 521-amino acid protein (accession number: MH683054) (Fig. 3B). Multiple sequence alignment identified a conserved bHLH–MYC_N domain and an HLH domain in *TabHLH1* (pfam 14215 and pfam 00010) using the Pfam online tool (http://pfam.xfam.org/search/sequence). Based on these results, we deduced that *TabHLH1* was a bHLH TF.

Subcellular localization showed that the yellow fluorescent protein (YFP) (control group; empty vector) was distributed uniformly throughout the cell, whereas TabHLH1-YFP fluorescence (treatment group; TabHLH1-YFP vector) was observed in the nucleus. This was confirmed using the positive control nuclear stain 4′,6-diamidino-2-phenylindole (DAPI) (Fig. 3C).

### Biochemical analyses of the main polyphenol compounds in *T. antungense* and expression profiling of *TabHLH1*

**Fig. 4:**
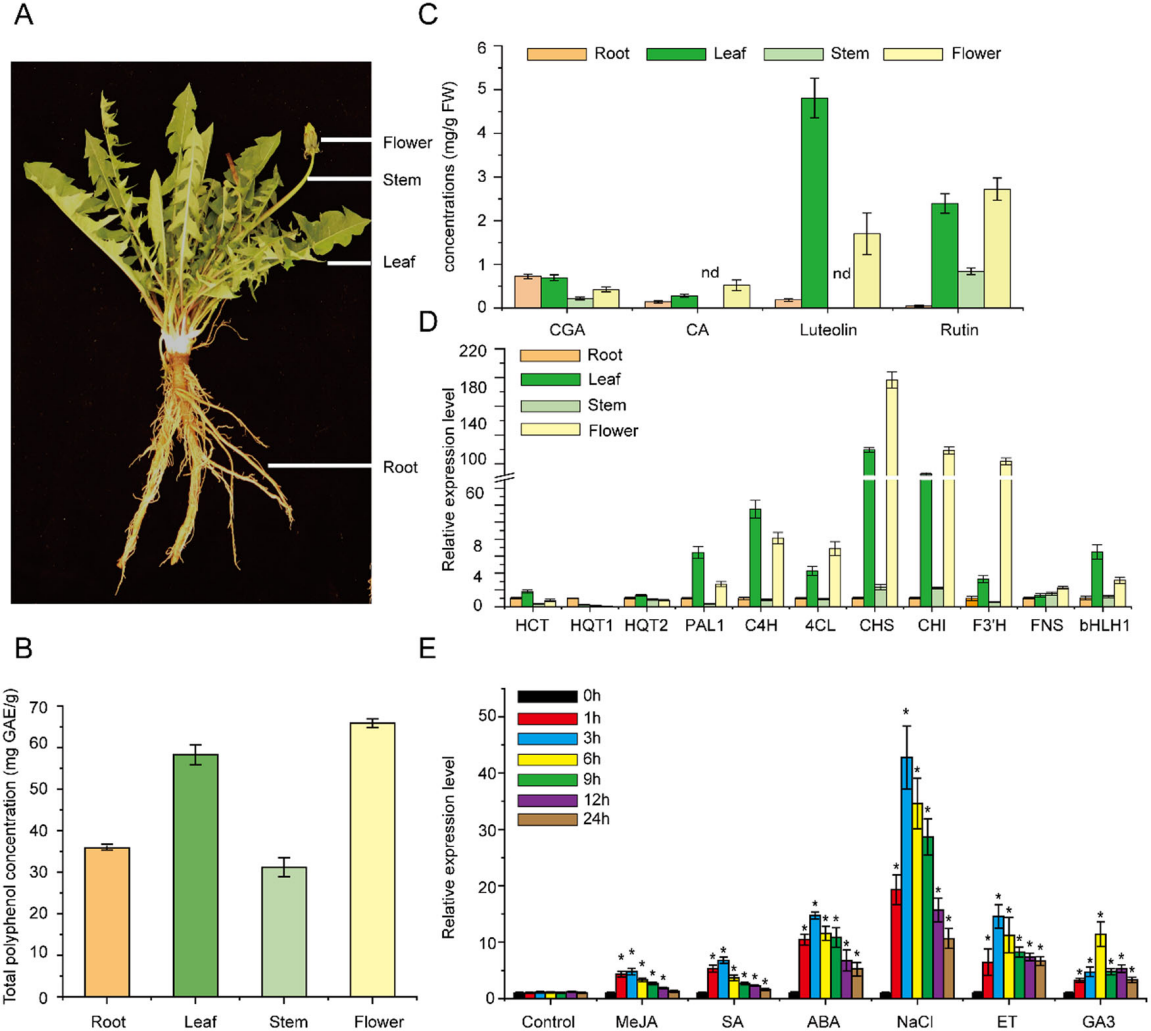
Basic biochemical analyses of *T. antungense* and the TabHLH1 elicitor response. **A** Different tissues (leaves, roots, flowers, and stem) of wild *T. antungense*. **B** Total polyphenol concentrations in different tissues. **C** CGA, CA, luteolin, and rutin concentrations in different tissues. nd: not detected. **D** The expression levels of polyphenol biosynthesis pathway enzyme genes (PAL, 4CL, C4H, HCT, HQT1, HQT2, CHS, CHI, FNS, F3′H) and TabHLH1 TF in different tissues of *T. antungense*. The expression levels of different genes in roots were set to 1. **E** The effects of different hormones on TabHLH1 gene expression levels. Treatment with 100 μM methyl jasmonate (MeJA), 100 μM salicylic acid (SA), 100 μM abscisic acid (ABA), 100 μM gibberellin (GA3), 100 μM ethylene (ET), or 500 μM NaCl for different time points (0, 1, 3, 6, 9, 12, 16, and 24 h). The expression levels of *TabHLH1* at 0 h were set to 1. The *TaActin* gene was selected as an internal reference in this article

The total phenolic concentrations of *T. antungense* in different tissue samples ranged from 32.37–66.23 mg GAE/g, and the highest concentrations of total phenolics were found in flowers, followed by leaves, roots, and stems (Fig. 4A, B). The four phenolic acids (CGA, CA, rutin, luteolin) in different tissues of *T. antungense* showed significant differences. CGA concentrations were highest in the roots, followed by flowers, stems, and leaves; CA concentrations were highest in the flowers, followed by leaves and flowers; rutin concentrations were highest in roots and flowers, followed by leaves and stems; luteolin concentrations were highest in leaves, followed by flowers and roots, while CA and luteolin were not detected in stems (Fig. 4C).

To study the correlation between key polyphenol biosynthesis enzyme genes and TabHLH1 TF in different tissues, one-year-old wild flowering *T. antungense* was used for expression analysis by qRT-PCR. The results showed that HQT1 was highly expressed in roots; CHS, CHI, 4CL, FNS, and F3′H were highly expressed in flowers followed by leaves; HCT, PAL, HQT2, and C4H were highly expressed in leaves followed by flowers. For TabHLH1, the expression level was highest in leaves, followed by flowers, roots, and stems (Fig. 4D).

To study the effects of various biotic and abiotic stresses on the expression level of *TabHLH1*, NaCl, MeJA, salicylic acid (SA), abscisic acid (ABA), ethephon (ET), and gibberellic acid (GA3) treatments were performed at different time points in *T. antungense* leaves, and then qRT-PCR was used for expression analysis. Under NaCl treatment (500 ng/mL), *TabHLH1* expression increased more than 40-fold in 3 h, indicating that *TabHLH1* was most responsive to NaCl treatment. After 3 h of ABA treatment, the expression level of *TabHLH1* reached 13-fold and had the same tendency as its expression under NaCl treatment. The expression level of *TabHLH1* was also affected by 100 μM MeJA; *TabHLH1* expression in leaves increased at 3 h and then slowly declined to approximately 1.3-fold at 24 h. Similar results were obtained with SA, ET, and GA3 hormone treatments (Fig. 4E).

### Identification of transgenic plants

**Fig. 5:**
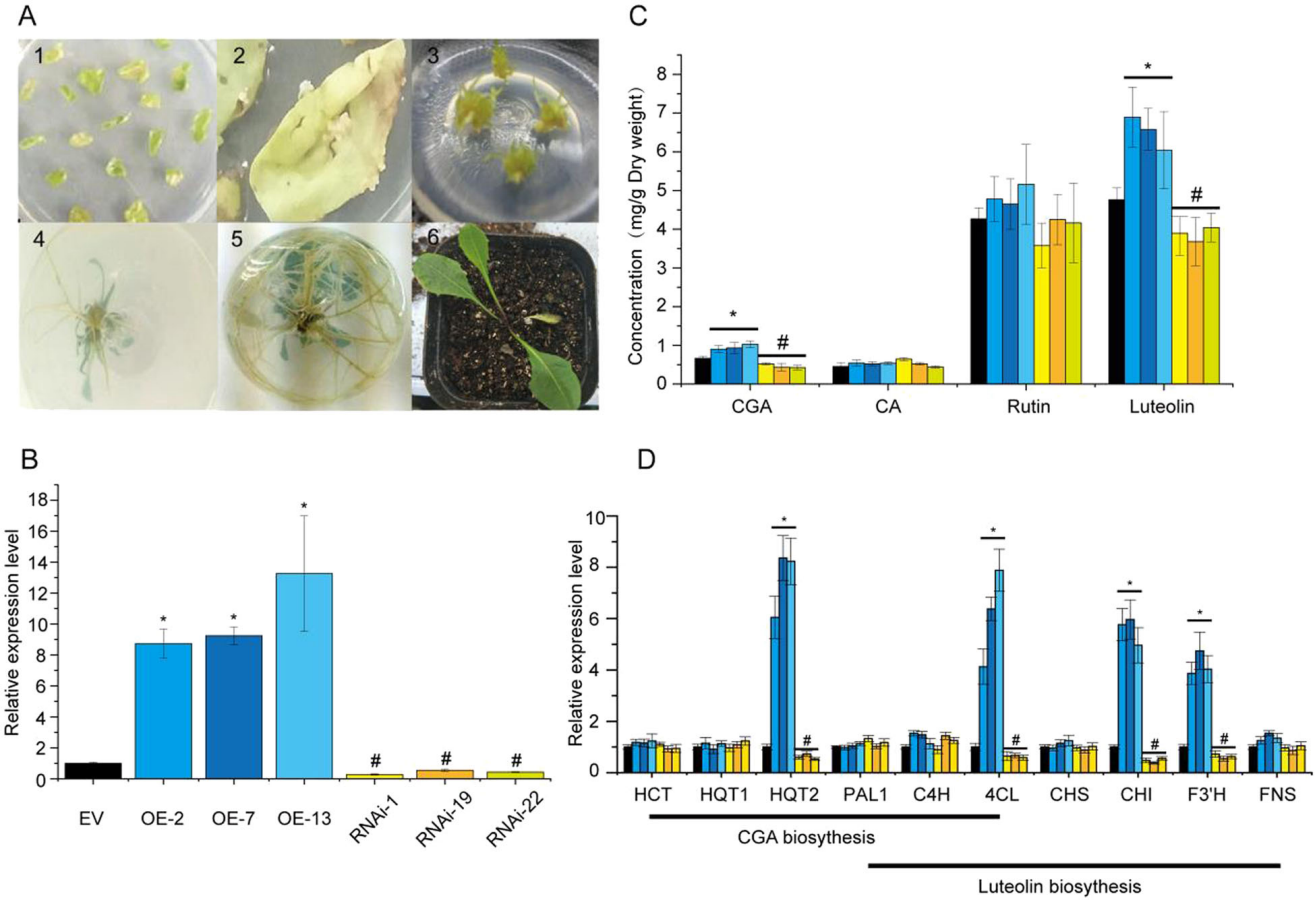
Generation of *T. antungense* transgenic lines. **A***Agrobacterium tumefaciens*-mediated transformation generated transgenic *T. antungense*; (1) callus induction for treatment with recombinant Agrobacterium GV3101 carrying the TabHLH1-overexpressing construct, (2) transgenic callus, (3) shoot induction, (4) root induction, (5) well-rooted transgenic plant, (6) transplantation of transgenic plants. **B** Expression level of TabHLH1 in different transgenic lines. **C** Determination of polyphenol concentrations, including those of chlorogenic acid (CGA), caffeic acid (CA), rutin, and luteolin, by HPLC in *T. antungense* transgenic lines. **D** Expression levels of the key enzyme genes in the CGA and luteolin biosynthesis pathways in control and TabHLH1 overexpression transgenic lines

To evaluate the regulatory mechanism of *TabHLH1* in *T. antungense* polyphenol biosynthesis, the overexpression vector pRI101-TabHLH1-YFP was constructed and transformed into *T. antungense* leaves according to Liu et al. (2018)^14^. Seventeen independent transgenic lines were identified using p35SF as the forward primer (according to the 35S promoter sequence) and TabHLH1R as the reverse primer through genomic PCR (Table S1). Three transgenic lines (TabHLH1-OE2, TabHLH1-OE7, and TabHLH1-OE13) with higher expression levels of OE-TabHLH1 than the control lines were selected for further experiments (Fig. S3 and Fig. 5A). At the same time, three RNAi-TabHLH1 transgenic lines (RNAi-1, RNAi-19, and RNAi-22) containing pCAMBIA1300-35S-TabHLH1 were identified according to Liu et al., 2019^18^. qRT-PCR showed that *TabHLH1* expression levels in the OE-TabHLH1 transgenic lines were significantly higher than those in the wild-type (WT) line, while the expression levels of RNAi-TabHLH1 were lower than those in the WT transgenic lines (Fig. 5B).

### Role of TabHLH1 in polyphenol biosynthesis

The concentrations of CGA, CA, rutin, and luteolin in the *TabHLH1* transgenic lines were determined by high-performance liquid chromatography (HPLC). CGA concentrations increased in the transgenic lines compared to those in the control lines (0.73 ± 0.054 mg/g dry weight [DW]), with the highest concentration in OE-7 (1.18 ± 0.113 mg/g DW) and the lowest in RNAi-19 (0.34 ± 0.141 mg/g DW). There were no significant differences in CA and rutin concentrations between the transgenic and control lines (Fig. 5C). Luteolin concentrations in OE-13 (7.07 ± 0.687 mg/g DW) increased compared to those in the control lines (4.83 ± 0.345 mg/g DW) and were lowest in RNAi-19 (3.84 ± 0.441 mg/g DW). To identify the CGA and luteolin biosynthetic genes regulated by *TabHLH1*, the expression levels of key enzyme genes in the CGA and luteolin biosynthesis pathways in *T. antungense* were first determined. *Ta4CL*, *TaHQT2*, *TaCHI*, and *TaF3*′*H* were all upregulated in the TabHLH1-overexpression lines to various degrees. *TaHQT2* expression was the most significantly increased among the four upregulated genes (Fig. 5D).

### In vivo and in vitro evaluation showed that TabHLH1 increased *TaHQT2* and *Ta4CL* expression

**Fig. 6:**
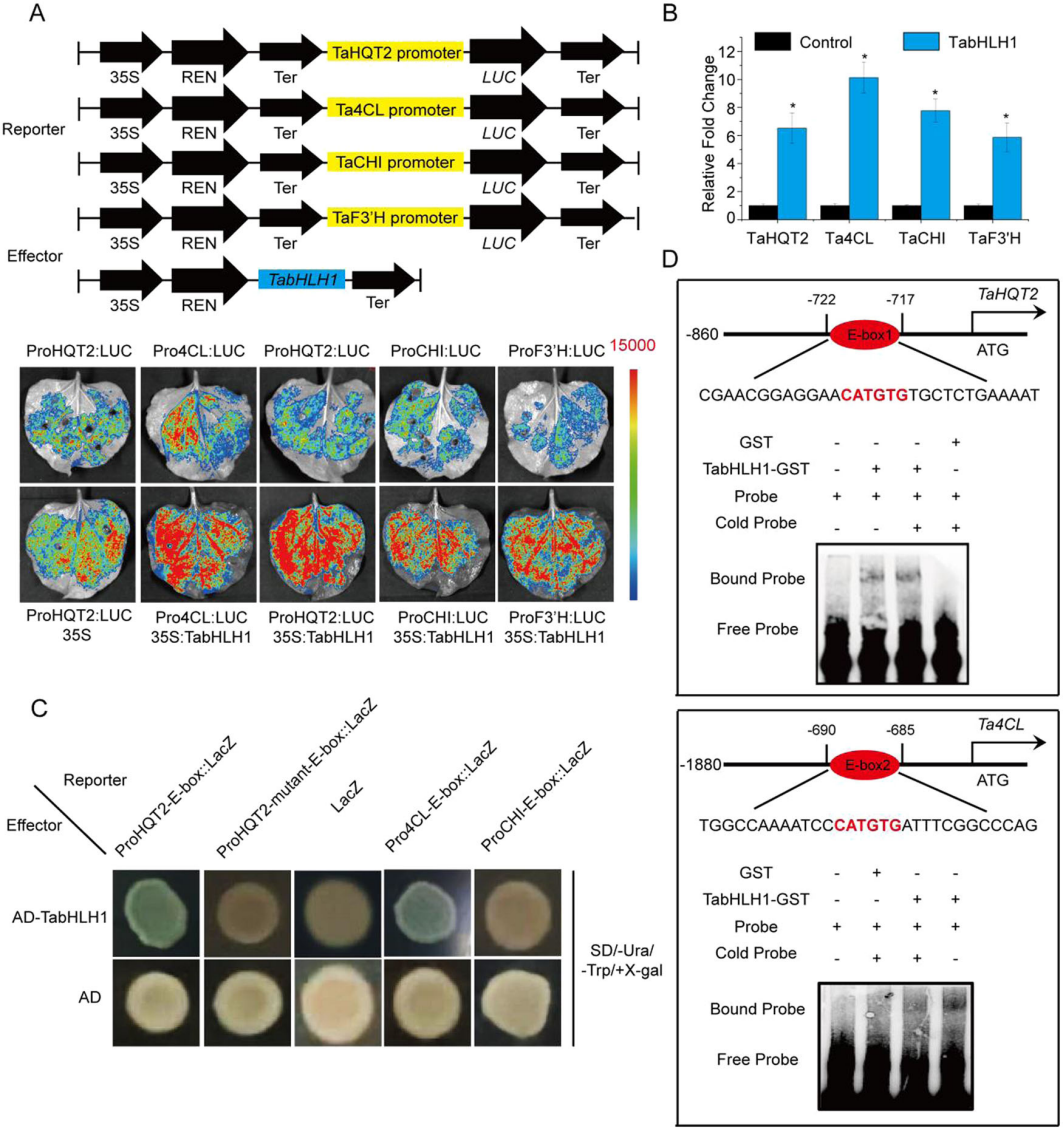
Dual-LUC and EMSAs proved that *TabHLH1* activates the expression of *Ta4CL, TaHQT2, TaCHI,* and *TaF3*′*H*. **A** Dual‐LUC assays indicating that TabHLH1 increases the expression levels of *Ta4CL*, *TaHQT2*, *TaCHI*, and *TaF3*′*H*. **B** The relative fold change of LUC/REN (Renilla) indicates that the expression levels of *Ta4CL*, *TaHQT2*, *TaCHI*, and *TaF3*′*H* can be activated by TabHLH1. **C** Y1H assay results indicated that TabHLH1 directly binds to *Ta4CL* and *TaHQT2* in yeast cells. The CATGTG motif has not been found in the promoter of *TaF3*′*H*. **D** EMSA results indicate that TabHLH1 directly binds to the E-box elements (CATGTG) from the *TaHQT2* and *Ta4CL* promoters. The cold-probe sequences were the same as the labeled probes but without biotin labeling

The gene promoters of *TaPAL*, *TaC4H*, *Ta4CL*, *TaHCT*, and *TaHQT1/2* from the CGA biosynthetic pathway all contain bHLH cis-acting elements^33^. Herein, dual-luciferase (LUC) assays were performed to investigate whether *TabHLH1* increased the expression of these genes. Reporter and effector vector construction are shown in Fig. 6A. Fluorescence analysis indicated the intensity of gene expression (Fig. 6A). Of the six examined genes, the ratio of LUC/Renilla (REN) was detected only for *Ta4CL* and *TaHQT2* and was significantly higher than that of the control, with a 6.26- and 10.08-fold increase, respectively (Fig. 6B). Furthermore, TabHLH1 binding sites (bHLH-responsive cis-elements) were detected using Y1H assays and electrophoretic mobility shift assays (EMSAs). TabHLH1 directly combined with the *TaHQT2* and *Ta4CL* promoters through the E-box motif (CATGTG) (Fig. 6C, D). These results indicated that *TabHLH1* directly increased *TaHQT2* and *Ta4CL* gene expression, thereby modulating CGA accumulation.

### TabHLH1 increased luteolin biosynthesis

Luteolin is also produced in plants using phenylalanine as a precursor^16^. Hence, we measured luteolin concentrations in the transgenic lines. Luteolin concentrations were significantly higher in the OE-TabHLH1 transgenic line and lower in the RNAi-TabHLH1 line than in the control groups (Fig. 5C). In addition, two of the luteolin biosynthetic pathway genes, *TaCHI* and *TaF3*′*H*, were strongly upregulated in the OE-TabHLH1 line, while *TaCHI* and *TaF3*′*H* were significantly downregulated in the RNAi-TabHLH1 line compared to that in the control groups (Fig. 5D). Dual-LUC assays showed that *TabHLH1* significantly increased *TaCHI* (harboring the CATGTG motif in their promoter) and *TaF3*′*H* (without the CATGTG motif) expression levels. The ratio of LUC/REN increased by 7.94-fold for *TaCHI* and 6.11-fold for *TaF3*′*H* compared to that in the control groups (Fig. 6B). However, Y1H assay results indicated that the E-box (CATGTG motif) in the *TaCHI* promoter cannot directly interact with *TabHLH1* (Fig. 6C). Together, these results indicated that *TabHLH1* increased both CGA and luteolin concentrations in *T. antungense* transgenic lines.

## Discussion

### Basic information on the polyphenols in *T. antungense*

In different tissues of *T. antungense*, the total polyphenol concentrations are highest among flowers, followed by leaves, roots, and stems, showing the same tendency as in *T. mongolicum*^4^. Total polyphenols, including chlorogenic, caffeic, quinic, caffeoylquinic, ferulic, cinnamic, caftaric, benzoic, vanillic, protocatechuic, gallic, cumaric acid, chrysoeriol, and vanillin, have been found in *Taraxacum* species^1^. Flowers and leaves have higher polyphenol concentrations than roots and stems. The CGA and CA levels in *T. mongolicum* were generally lower than those in *T. antungense*, while luteolin levels were significantly higher^4^. The possible reasons are various factors, including growth environments, sample extraction, and HPLC methods, which together cause significant differences in CGA, CA, rutin, and luteolin concentrations in *T. antungense* and closely related species^4,21,37^. The relationship among the four compounds and total polyphenol concentrations should be further studied.

### TabHLH1 potentially regulates polyphenols in *T. antungense*

First, the HQT2 gene promoter was used as bait to screen functional TFs. CGA is widely distributed in many plants, such as *T. antungense*, *L. japonic*a, *Solanum lycopersicum*, and *Solanum tuberosum*^20,21,38^. In the CGA biosynthesis pathway, key enzyme genes, such as PAL, C4H, 4CL, and HCT/HQT, are widely reported^14,20^. However, the regulation of CGA biosynthesis targeting the key enzyme HQT has not been previously reported^20,21^. Gene promoters are the core link between TFs and structural gene expression^39–41^. Through analysis of the *TaHQT2* promoter (four CANNTG motifs), it is speculated that *TaHQT2* is regulated by bHLH TFs (Fig. S1 and Table S2)^33,41^. Herein, using proHQT2 as a probe, we first obtained a bHLH TF named TabHLH1 through Y1H assays.

Second, coexpression analysis can be used to screen and preliminarily identify the correlation between transcription factors and key enzyme gene expression^34,40^. Under ABA treatment, *SmbZIP1* and salvianolic acid biosynthesis pathway gene expression levels significantly increased, and coexpression analysis showed a highly linear relationship^40^. By analyzing *TabHLH1* together with polyphenol biosynthesis pathway gene expression levels in *T. antungense* different tissues, it can be concluded that TabHLH1 potentially regulates the expression of key enzyme genes in polyphenol biosynthesis and thus affects the concentration of polyphenols in *T. antungense*^32,33^. Therefore, polyphenol analysis, Y1H assays, and coexpression analysis provide a theoretical basis for screening related TFs for designing molecular breeding strategies to improve *T. antungense* quality.

### TabHLH1 increased CGA biosynthesis in *T. antungense*

In this study, full-length *TabHLH1* was isolated and cloned from *T. antungense* and showed the highest identity with the *Gerbera hybrid* cultivar *GhbHLH1*. Both *GbbHLH1* and *GhbHLH1* play a vital role in the regulation of anthocyanin and dihydroflavonol accumulation^37,42,43^. In plants, anthocyanin and CGA have the same precursor and share the first three key enzymes^41^. In this study, salt stress significantly increased *TabHLH1* gene expression levels, with the same tendency as that of *GhbHLH1*, which is consistent with earlier findings^42^. However, under ABA treatment, *TabHLH1* expression increased more than 10-fold, which has not been reported in *GbbHLH1*^42^. A possible reason for this finding may be species evolution and functional redundancy in multiple gene families^31^. TabHLH1 is located in the nucleus, similar to other nucleus-localized bHLH TFs, such as AaMYC2-Like and MdMYC2^44,45^. Therefore, TabHLH1 plays a vital role in hormone and salt stress signal regulation, which ultimately assists plants in their response to a variety of biotic and abiotic stresses in their natural environment^14,26^. The relationship among hormones, salt stress signal regulation, and TabHLH1 expression should be further studied.

Multiple TFs, such as SmbHLH37 and SmbHLH53, increase polyphenol concentrations through PAL or other key enzymes^32,33^. By analyzing the CGA concentrations in TabHLH1 transgenic lines (OE-7 and RNAi-19), CGA concentrations in the OE-7 transgenic line increased nearly 63.6% compared to that in the WT, while CGA concentrations in RNAi-19 decreased to 53.2%, suggesting that *TabHLH1* significantly increased CGA concentrations in *T. antungense*. TaHQT2 was identified as the key enzyme that can directly synthesize CGA in previous studies^21^. In this study, *Ta4CL* and *TaHQT2* gene expression levels were positively correlated with the CGA concentration. Furthermore, dual-LUC and EMSA demonstrated that TabHLH1 bound directly to the *TaHQT2* promoter region. These results together demonstrated that *TaHQT2* was a target of *TabHLH1*; thus, TabHLH1 played a positive role in regulating CGA biosynthesis.

p-Coumarin-CoA is a precursor compound for the synthesis of downstream phenolic acids, flavonoids, and anthocyanins^7,42^. 4CL participates in the front-end enzymatic reaction of polyphenol biosynthesis and directly catalyzes the biosynthesis of p-coumarin-CoA (Fig. 1). In this study, *TabHLH1* increased the expression level of *Ta4CL*, thereby regulating metabolic flow in plants to accumulate polyphenols, which is consistent with CsbHLH1 directly activating the *Cs4CL* promoter^44,46,47^. Dual-LUC, Y1H, and EMSA demonstrated that TabHLH1 bound directly to the *Ta4CL* promoter region. These results suggested that *Ta4CL* was another target of *TabHLH1*.

### TabHLH1 increased *CHI* and *F3*′*H* gene expression levels in vivo to promote luteolin biosynthesis in *T. antungense*

Flavonoid biosynthesis regulation has been studied in-depth, particularly in the medicinal plant *S. miltiorrhiza*^19,33,48^. Functional genes, including key enzyme genes and numerous TFs, are required for luteolin biosynthesis^29,44^. In this study, *TabHLH1* significantly increased luteolin concentrations (OE-7 increased to 1.46-fold, and RNAi-19 decreased to 0.78-fold) in transgenic lines compared to that in the WT. In addition, concentrations of the key luteolin biosynthesis enzymes *TaCHI* and *TaF3*′*H* were directly influenced by overexpression/RNAi of *TabHLH1*, suggesting that TabHLH1 may interact with *TaCHI* and *TaF3*′*H*. Dual-LUC results showed that TabHLH1 increased the expression levels of two pathway genes involved in luteolin biosynthesis. However, TabHLH1 binds indirectly to the promoters of *TaCHI* and *TaF3*′*H* (Fig. 6C). The possible reason may be that TabHLH1 interacts with other proteins, such as MYB partners, which can directly interact with *TaCHI* and *TaF3*′*H*. These results indicated that TabHLH1 was positively involved in regulating luteolin biosynthesis and metabolic flow, and the direct target was *Ta4CL*, while the potential targets were *TaCHI* and *TaF3*′*H*. The MYB-bHLH-WD40 complex often increases the expression level of flavonoid biosynthesis genes to control the production of anthocyanins^49^. *CsbHLH1* (*CsMYC1*) interacts with *CsbHLH42*, *CsWD40*, *CsMYB60* and itself to regulate flavonoid biosynthesis in cucumber^47^. In *T. antungense*, further studies should focus on proteins that interact with *TabHLH1* to regulate polyphenol biosynthesis.

**Fig. 7:**
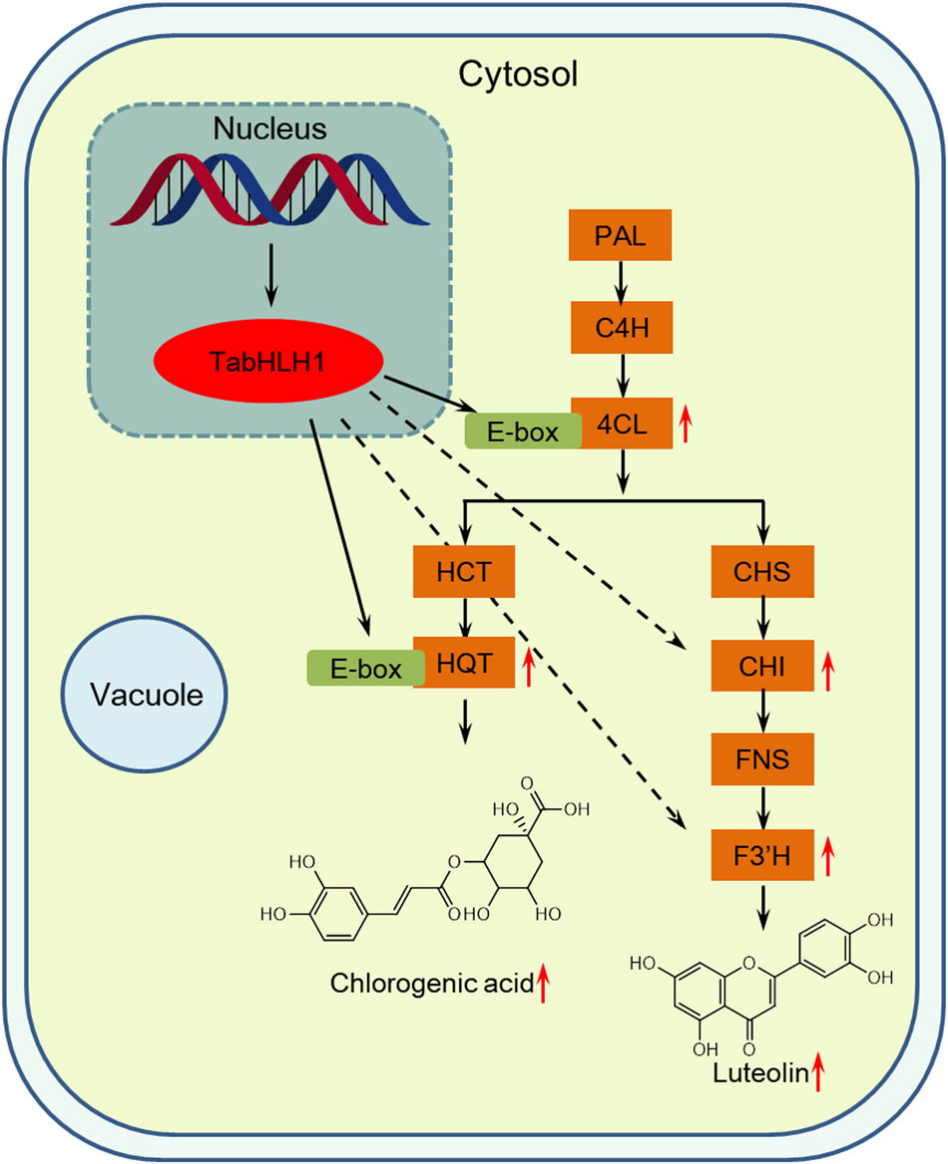
Proposed model of the role of TabHLH1 in regulating polyphenol biosynthesis. Using the HQT2 promoter E-box region as a probe, a bHLH transcription factor (named *TabHLH1*) was obtained by yeast one-hybrid screening. TabHLH1 significantly promoted the expression levels of key enzyme genes in the polyphenol biosynthesis pathway, including 4CL, HQT, CHI, and F3′H, thus increasing CGA and luteolin accumulation

Based on these results, a functional model for the role of TabHLH1 in polyphenol biosynthetic regulation in *T. antungense* is provided (Fig. 7). In summary, TabHLH1 increased *TaHQT2* and *Ta4CL* expression levels, leading to increased CGA concentrations. This is the first dandelion bHLH protein identified as being involved in CGA pathway regulation. In addition, TabHLH1 promoted the expression of luteolin biosynthesis genes (*TaCHI* and *TaF3*′*H*) to increase the accumulation of luteolin. These studies provide new insights into the role of TabHLH1 in the regulation of polyphenol biosynthesis. In addition, these findings lay the foundation for further exploration of the molecular mechanisms and potential functional genes of secondary metabolite biosynthesis in *T. antungense*.

## Materials and Methods

### Plant materials

*T. antungense* biomaterials were collected and transplanted in the greenhouse of our laboratory, as previously reported^34^. *N. benthamiana* and *T. antungense* seeds were sown in substrate/vermiculite (3/1)-admixture soil and transplanted in pots for 4–6 weeks for hormone treatment experiments. Plants were maintained at a constant temperature of 25 °C under 16/8 h light/dark cycles for use in transient expression analysis^14^.

### TaHQT2 promoter cloning and Y1H screening

The CTAB method was used to extract dandelion plant DNA, and RNase was used to remove the remaining RNA. Fusion primers and nested integrated PCR were used to obtain the *TaHQT2* 5′-end promoter^47,50^. All primers are listed in Table S1. PlantCARE (http://bioinformatics.psb.ugent.be/webtools/plantcare/html/) was used to analyze the promoter cis-element sequences.

The *TaHQT2* promoter (four E-boxes located from –691 bp to –806 bp, Fig. S1) was constructed in pABAi to create the pAbAi-proTaHQT2 recombinant vector using the *BamH* I and *Hind* III restriction sites. *BstB*I was used to digest recombinant plasmids, linearized pAbAi-proTaHQT2 plasmids were transformed into the yeast strain (Y1H), and then, the resulting strains were tested on SD/–Ura media containing aureobasidin A (AbA) at concentrations ranging from 100–500 ng/mL.

A Y1H cDNA library of *T. antungense* was constructed using the Matchmaker™ one-hybrid library construction & screening kit PT3529-1 (PR732190, Takara Biomedical Technology Co., Ltd., Beijing, China). Yeast recombinant vector (GAL4-AD-sec) was extracted from the primary library and transformed into Y1H containing pAbAi-proTaHQT2. After rescreening on SD/-Leu-Ura medium with higher AbA concentrations than listed above, positive pGADT7-sequence yeast strains were used for sequencing analysis. The PCR thermocycler program used was as follows: 94 °C for 10 min, 40 cycles of denaturation, annealing, and extension (94 °C for 30 s, 50 °C for 45 s, and 72 °C for 90 s, respectively), and a final extension at 72 °C for 10 min.

### Bioinformatics analysis and isolation of TabHLH1

A bHLH TF protein isolated from *T. antungense*, which was designated *TabHLH1*, was found to interact with the E-boxes of *TaHQT2*. The gene sequence was compared to *Taraxacum kok-saghyz* Rodin (accession number: GWHAAAAM043215)^36^. The complete coding sequence was obtained using homologous cloning. The target cDNA fragment was connected to the 18 T vector (Takara Biomedical Technology Co., Ltd., Beijing, China) for sequencing analysis. BLAST alignment (http://www.ncbi.nlm.nih.gov/BLAST/) was used to search for orthologs. ClustalX (version 1.81) was used for multiple sequence alignment through the neighbor-joining method using 1000 repetitions. A molecular phylogenetic tree was constructed using the MEGA program (version 8.0)^14^.

### Elicitor treatment and subcellular localization of TabHLH1

Methyl jasmonate (MeJA), salicylic acid (SA), ethephon (ET), gibberellin (GA), and abscisic acid (ABA) purchased from Sigma-Aldrich (Shanghai, China) were directly dissolved in distilled water at a final concentration of 100 mM. NaCl (Aladdin, Shanghai, China) was dissolved at a concentration of 500 mM, and distilled water was used as a control. Two-month-old well-grown *T. antungense* transgenic and WT plants were chosen for different treatments. The above elicitors were filter-sterilized through a 0.45 μm filter membrane (Pall Corporation, NY, USA) and added to the cultures at a final concentration of 100 μM. Tissues from different parts of the plants were collected after 0, 1, 3, 6, 9, 12, 16, and 24 h of treatment.

To identify the in vivo subcellular location of TabHLH1, the coding sequence of *TabHLH1* was fused with a reporter sequence. The complete coding sequence of *TabHLH1* (without the TAG stop codon), including the restriction sites *Nde* I (in the 5′-end) and *Sal* I (in the 3′-end), was amplified and subcloned into the pMD19-T simple vector (Takara Biomedical Technology Co., Ltd., Beijing, China). The plasmid pRI101-YFP (containing the yellow fluorescent protein gene) was double-digested using the same enzymes to create a recombinant vector termed pRI101-TabHLH1-YFP, and the insert was sequenced using the 35S (forward) and TabHLH1R (reverse) primers (Fig. S2 and Table S1). The fused recombinant expression plasmid was transformed into *N. tabacum*. pRI101-YFP was used as a control to perform the transient expression assay^21,40^.

### Transformation of *T. antungense*

The pCAMBIA1300-35S-X (restriction sites: *BamH* I/*Spe* I for sense and *Kpn* I/*Sac* I for antisense sequences) RNAi expression vector was used for RNAi-*TabHLH1* recombinant plasmid creation^21^. The SPLRNAi gene was used as intron X. The middle region of *TabHLH1* (631–839 bp) was used for vector construction (nonconserved region). Both pCAMBIA1300-35S-TabHLH1 and pRI101-TabHLH1-YFP were transformed into *Agrobacterium tumefaciens* strain GV3101. pCAMBIA1300-35S-X and the pRI101-YFP vector were used as controls. After positive identification, *Agrobacterium* harboring different recombinant plasmids were used for injection into plants. Following a previously published protocol^14^, *Agrobacterium* infection was used for genetic transformation to obtain *T. antungense* transgenic plants.

### Analysis of gene expression profiles

Different tissues or transgenic lines of *T. antungense* were used for total RNA extraction, followed by cDNA synthesis, which was performed following the abovementioned methods^50,51^. qRT-PCR was performed using gene-specific primer pairs for *PAL*, *C4H*, *4CL*, *HCT*, *HQT2*, *CHS*, *CHI*, and *F3*′*H* (Supplemental Table S1) using three technical replicates. Based on the 2^−∆∆Ct^ method, qRT-PCR was performed, and relative expression levels were calculated using β-actin as a reference gene^14^.

### Measurement of polyphenol concentrations by HPLC

HPLC was used to investigate the concentrations of four polyphenols (CGA, CA, rutin, and luteolin) in the *T. antungense* plant materials from the control groups and transgenic lines (Table S3). For transgenic lines, 3-month-old whole plants (containing roots and leaves) were dried and dehydrated at −20 °C to constant weight, ground into powder, and then used as samples. Samples were ultrasonically extracted for polyphenol compounds and passed through a 0.22 μm filter membrane for HPLC, as previously described^14,23^. HPLC conditions for polyphenol detection were as described in the previous reports^34^.

### Measurement of total phenolic concentrations

The total phenolic concentrations of *T. antungense* in different tissues were extracted with Folin-Ciocalteu reagent as previously reported^4,5^. Then, 500 µL of *T. antungense* extract was added to 1.5 mL FC reagent (0.2 mg/mL) and mixed. Two milliliters of 7.5% Na_2_CO_3_ reagent and 2 mL distilled water were added. Then, the mixture was incubated at 25 °C for 1 h (in the dark). The absorbance of the mixture was recorded at 727 nm, and 60% methanol was used as a control. The total phenolic concentrations of *T. antungense* samples were calculated according to milligrams of gallic acid equivalents per gram dry weight of the sample (mg GAE/g)^4^

### Dual-LUC assay

The pCAMBIA2300^+^-TabHLH1 vector acted as an effector and was transferred into *A. tumefaciens* strain GV3101 (pCAMBIA2300^+^*-*vector was used as a control). The promoters of key enzyme genes, including *Ta4CL*, *TaHQT2, TaCHI*, and *TaF3*′*H*, were cloned separately into the pGreen0800-rec plasmid. The pGreen0800-promoter recombinant vectors, separately with the helper vector pSoup19, were cotransformed into GV3101. The *Renilla* vector was used as an internal control. Both the reporter and effector strains were mixed in equal proportions (3 mL each), slowly cultivated for 2 h, and injected into the leaves of 2-month-old *N. benthamiana*. After incubation in the dark for 2–3 days, commercial dual-LUC reaction reagents (Promega Biomedical Technology Co., Ltd., Beijing, China) were used to perform dual-LUC assays on leaf samples^18^. Three biological replicates were measured for each sample.

### Y1H assay

The Y1H assay was performed differently from the Y1H screening and used the pB42AD/pLacZ system, which has been previously described^40^. The full-length TabHLH1 ORF fragment was amplified, sequenced, and cloned into the effector plasmid (pB42AD). For the reporter plasmid pLacZ, a triple tandem copy of the E-box (NNNN**CATGTG**NNNN) motif near 4 bp from every promoter (TaHQT2, Ta4CL, TaCHI) was inserted by using *EcoR* I and *Xho* I as restriction endonuclease sites. After identification by sequencing, recombinant effector plasmids (pB42AD-TabHLH1) and recombinant reporter plasmids (pLacZ-TaHQT2, pLacZ-Ta4CL, pLacZ-TaCHI) were cotransformed into yeast strain EGY48a. Transformants were cultivated on SD/-Ura/-Trp medium for 2 d and then transferred (by using distilled water-diluted 1000-fold) to SD/-Ura/-Trp medium with 5-bromo-4-chloro-3-indolyl-β-D-galactopyranoside (X-gal) for another 1–2 d. Empty pB42AD and pLacZ plasmids were used as negative controls and cotransformed into EGY48a strains.

### EMSAs

The complete sequence of *TabHLH1* was inserted into the *BamH* I and *Sal* I sites of the pGEX4T-1 plasmid and then transformed into *Escherichia coli* (BL21 or DE3 strain). Isopropyl-D-thiogalactoside was used to induce recombinant protein expression overnight (16 h), and the GST-tagged protein purification kit (Transgen Biotech Co., Ltd., Beijing, China) was used to purify recombinant proteins. Biotin-labeled 5′- and 3′-ends of the *TaHQT2* promoter were synthesized by Shanghai Sangon Co. (Shanghai, China), and the two biotin-labeled primers were annealed to form double-stranded DNA fragments. The purified recombinant proteins and DNA fragments were incubated in 10× EMSA binding buffer (Beyotime Biotechnology Co., Ltd., Shanghai, China) at 25 °C for 30 min. DNA fragments without biotin labeling were used as an internal control. The DNA-protein complex was electrotransferred to a wet electromembrane and examined following the manufacturer’s instructions by using a chemiluminescent nucleic acid detection module kit (Beyotime Biotechnology Co., Ltd., Shanghai, China)^19,27,48,52^.

### Statistical analyses

Statistical comparisons were performed using SPSS v19.0 software. Error bars represent the SE of three biological replicates. All data are presented as the mean±standard deviation (SD). Statistical significance was assessed using Student’s *t* test (#: decrease, *: increase, *P* < 0.05) for all the experiments involved in this article (for the different tissues, transgenic lines, and control groups).

## Supplementary information

Supplemental material
