# Supplementary material for: The basic helix-loop-helix transcription factor TabHLH1 increases chlorogenic acid and luteolin biosynthesis in Taraxacum antungense Kitag
Source: Hortic Res. 2021 Sep 1;8:195. doi: 10.1038/s41438-021-00630-y (PMC8408231; doi:10.1038/s41438-021-00630-y)
Supplement: Supplementary file 1 — Supplemental material [file 41438_2021_630_MOESM1_ESM.docx]

**Fig. S1** promoter of TaHQT2


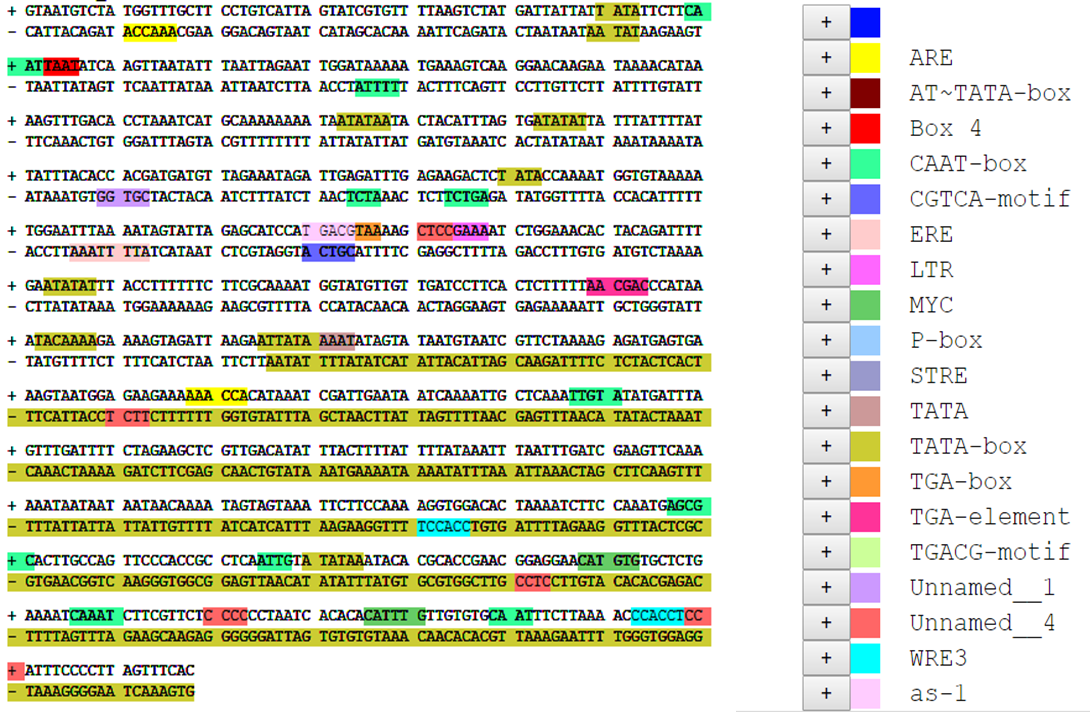


**Fig. S2** *Taraxacum antungense* cDNA library construction


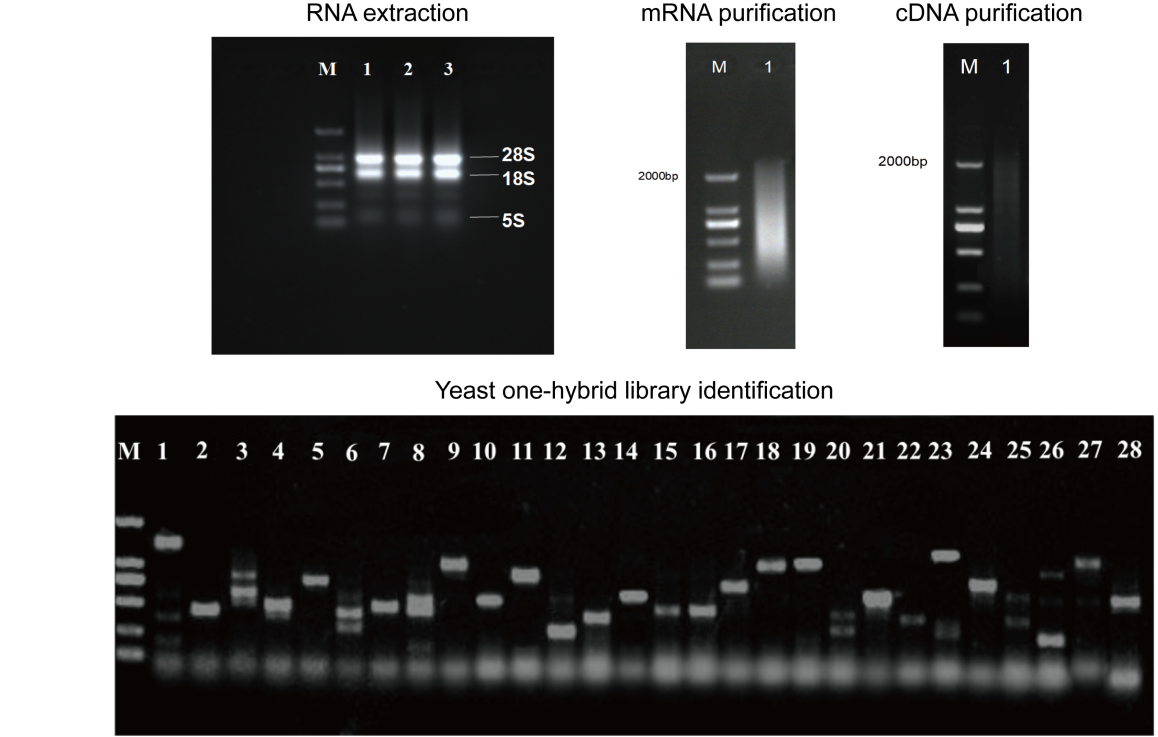


**Fig. S3** Vector construction and transgenic lines identification


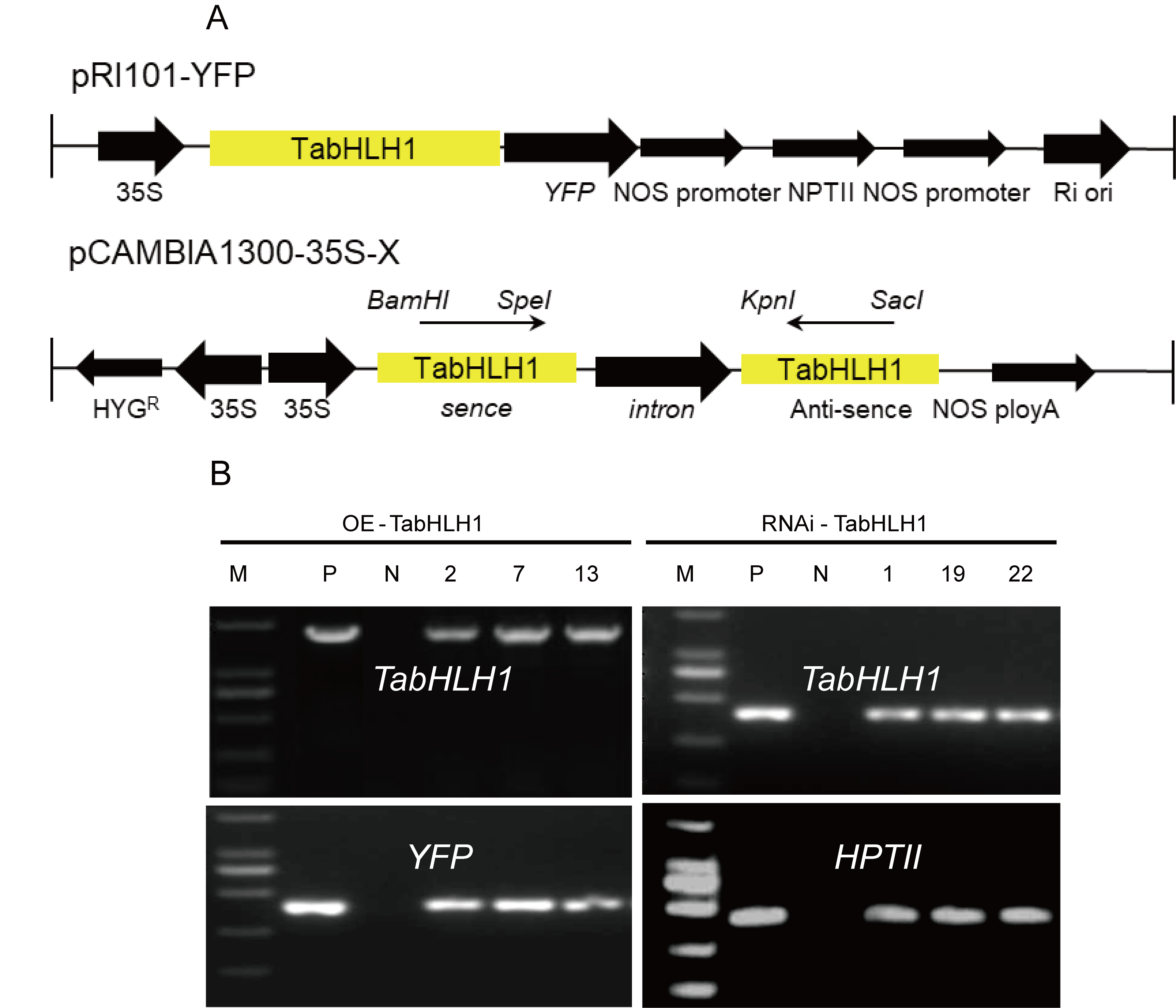


B:qRT-PCR expression of the yellow fluorescent protein (YFP) gene and TabHLH1-YFP fusion gene in the empty vector and the three *TabHLH1* overexpressing independent transgenic lines; qRT-PCR expression of HPTII and partial gene in the empty vector (EV) and three TabHLH1 RNAi independent transgenic lines.

**Table. S1** The list of primers used in this study

| function | Primer | Nucleotide Sequence |
| --- | --- | --- |
| FPNI-PCR  (Wang et al., 2011) | FP4(1st PCR primer) | 5'-GTA ATA CGA CTC ACT ATA GGG CAC GCG TGG TAG WGN AGW ANC AWA G-3' |
|  | FP7(1st PCR primer) | 5'-GTA ATA CGA CTC ACT ATA GGG CAC GCG TGG TNG ACG ASW GAN AWG AA-3' |
|  | FSP1(2nd PCR primer) | 5'-GTA ATA CGA CTC ACT ATA GGG C-3' |
|  | FSP2(3rd PCR primer) | 5'-ACT ATA GGG CAC GCG TGG T-3' |
| Promoter of TaHQT2 | SP1 | 5'- ATA GCT GCT TAG GAC ATT CT -3' |
|  | SP2 | 5'- CTG AAG GAG GAA TGA TGG -3' |
|  | SP3 | 5'- CAT CTT TTG ATC ACT ACC CAT -3' |
| pAbAi-proTaHQT2 | pHQT2F | 5'-CCA AAT GCA ATT GCATGTG CAT TTGG-3' |
|  | pHQT2R | 5'-TCG ACC AAA TGCACATG CAA TTG CAT TTG GAG CT-3' |
| pAbAi-pMutant-proTaHQT2 | pMHQT2F | 5'-CCA AAT GCA ATT GAAAAAA CAT TTGG-3' |
|  | pMHQT2R | 5'-TCG ACC AAA TGTTTTTTCAA TTG CAT TTG GAG CT-3' |
| TabHLH1 | bHLH1F | 5'-ATG GCT GCA ATG GAA AAC TTG-3' |
|  | bHLH1R | 5'-CTA GAA ACT TAC AGA ACT CCT-3' |
| pRI101- TabHLH1-YFP | TabHLH1F | 5'-CG CATATG ATG GCT GCA ATG GAA AAC TTG-3', NdeI site underlined |
|  | TabHLH1R | 5'-CG GTCGAC CTA GAA ACT TAC AGA ACT CCT-3' SalI site underlined |
| pCAMBIA1300-35s-X | RNAi-sence-TabHLH1F | 5'-CG CATATG CCA CAC ATT CTT GAT GAT GA-3', BamHI site underlined |
|  | RNAi-sence-TabHLH1R | 5'-CG ACTAGT ATA GAT TAC TCG GAC CAC TC-3' SpeI site underlined |
|  | RNAi-anti-sence-TabHLH1F | 5'-CG GGTACC ATA GAT TAC TCG GAC CAC TC -3' KpnI site underlined |
|  | RNAi-anti-sence-TabHLH1R | 5'-CG GAGCTC CCA CAC ATT CTT GAT GAT GA -3' SacI site underlined |
| AD-TabHLH1F | AD-TabHLH1F | 5'-CCA GAT TAC GCT CATATG ATG GCT GCA ATG GAA AAC TTG-3', NdeI site underlined |
|  | AD-TabHLH1R | 5'-CTC GAG CTC GAT GGATCC CTA GAA ACT TAC AGA ACT CCT-3', BamHI site underlined |
| p35SF | 35S-promoter | 5'-CTA TCC TTC GCA AGA CCC TTC-3' |
| T7-promoter | T7-promoter | 5'-TAA TAC GAC TCA CTA TAG GG-3' |
| M13F (-47) | M13F (-47) | 5'-CGC CAG GGT TTT CCC AGT CAC GAC -3' |
| M13R (-48) | M13R (-48) | 5'-AGC GGA TAA CAA TTT CAC ACA GGA-3' |
| β-actin | ACTIN R | 5'-AGC AGC TTC CAT TCC GAT CA-3' |
|  | ACTIN F | 5'-GGT TAC ATG TTC ACC ACC AC-3' |
| Biotin-labeled probe | HQT2F biotin-labeled probe | 5'-CGAACGGAGGAA**CATGTG**TGCTCTGAAAAT-3' with 3’ biotin-labeled |
|  | HQT2R biotin-labeled probe | 5'-ATTTTCAGAGCA**CACATG**TTCCTCCGTTCG-3' with 3’ biotin-labeled |
| Cold probe | Cold HQT2F probe | 5'-CGAACGGAGGAA**CATGTG**TGCTCTGAAAAT-3' |
|  | Cold HQT2R probe | 5'-ATTTTCAGAGCA**CACATG**TTCCTCCGTTCG-3' |
| Biotin-labeled probe | 4CLF biotin-labeled probe | 5'-TGGCCAAAATCC**CATGTG**ATTTCGGCCCAC-3' with 3’ biotin-labeled |
|  | 4CLR biotin-labeled probe | 5'- GTGGGCCGAAAT**CACATG**GGATTTTGGCCA -3' with 3’ biotin-labeled |
| Cold probe | Cold 4CLF probe | 5'- TGGCCAAAATCC**CATGTG**ATTTCGGCCCAC -3' |
|  | Cold 4CLR probe | 5'- GTGGGCCGAAAT**CACATG**GGATTTTGGCCA -3' |
| qPCR for TabHLH1 | qTabHLH1F | 5'-GCC ATC TTC TGG TCC ATT TCT AC-3' |
|  | qTabHLH1F | 5'-TCA TCG TCA TCC TCC TTC ATT TC-3' |
| qPCR for TaPAL1 | TaPAL1F | 5'-CCA ACA AAG CGG TCT CGT AT -3' |
|  | TaPAL1R | 5'-ATT GGT GGA GAG ACG CTG AC -3' |
| qPCR for TaC4H | TaC4HF | 5'-CTC GGA CTC GGT GTA CAG-3' |
|  | TaC4HR | 5'-CCT CAG GGT TGT TGG CTAG-3' |
| qPCR for Ta4CL1 | Ta4CLF | 5'-TGG CGC TAC CGT ACT CCTC-3' |
|  | Ta4CLR | 5'-CAA ATC AAC ACA TCC TCC-3' |
| qPCR for TaHCT | TaHCTF | 5'-AGT TTA CAT GTG GTG CGA CAT-3' |
|  | TaHCTR | 5'-TGT GTC ATT TGG AGT GGCC-3' |
| qPCR for TaHQT2 | qTaHQT2F | 5'-TGG CAG GGA GAA TGG ATA-3' |
|  | qTaHQT2R | 5'-AGT CAA CAG TCG GAG TAA GA-3' |
| qPCR for TaCHS | TaCHSF | 5'-CAG AAC TAG CAA CCG AAG GC -3' |
|  | TaCHSR | 5'-CCG TAA CAT CCC AAG AAG -3' |
| qPCR for TaCHI | TaCHIF | 5'-GAT TGG TGT GTA TTT GGA GG -3' |
|  | TaCHIR | 5'- GTA AGT CCC ATG TGC TTT CC -3' |
| qPCR for TaFNS | qTaFNSF | 5'- AGG TAT CTG AGT TGG CAA AC -3' |
|  | qTaFNSR | 5'-GGA AAG AGC AGC GGA CCC AC-3' |
| qPCR for TaF3’H | qTaF3’HF | 5'-GGA ACT ATC AGA AAT CAT AG -3' |
|  | qTaF3’HR | 5'-TTC AGG ATC TCT GTG TAT CG-3' |
| pB42AD-TabHLH1 | AD-TabHLH1F | 5'- GATTATGCCTCTCCCGAATTC ATG GCT GCA ATG GAA AAC TTG -3' |
|  | AD-TabHLH1R | 5'- AGAAGTCCAAAGCTTCTCGAG CTA GAA ACT TAC AGA ACT CCT -3' |
| *pLacZ*–proTaHQT2 | proTaHQT2F | 5'- GGAA**CATGTG**TGCTGGAA**CATGTG**TGCTGGAA**CATGTG**TGCT -3' |
|  | proTaHQT2R | 5'- AGCA**CACATG**TTCCAGCA**CACATG**TTCCAGCA**CACATG**TTCC -3' |
| *pLacZ*–proTa4CL | ProT4CLF | 5'- ATCC**CATGTG**ATTTATCC**CATGTG**ATTTATCC**CATGTG**ATTT -3' |
|  | Pro4CLR | 5'- AAAT**CACATG**GGATAAAT**CACATG**GGATAAAT**CACATG**GGAT -3' |
| *pLacZ*–proTaCHI | proCHIF | 5'- AACA**CATGTG**TTTGAACA**CATGTG**TTTGAACA**CATGTG**TTTG -3' |
|  | proTaCHIR | 5'- CAAA**CACATG**TGTTCAAA**CACATG**TGTTCAAA**CACATG**TGTT -3' |

**Table. S2** Cis-acting element of *proTaHQT2*

| cis-regulatory element | Position (strand) | Sequence | Function |
| --- | --- | --- | --- |
|  |  |  |  |
| ARE | -11 (-), 508(+) | TGGTTT | An essential condition element for anaerobic induction |
| TATA-box | -59(-), -173(+), -193(+), -260(+),-353(+), -422(+),-445(+),-454(-),-459(-),-548(-),-600(-), -727(-), -722(-), -730(+) | TATAATAAT | The core initiating element at about -30 upstream of the transcription start site |
| Box 4 | -71(+) | ATTAAT | Light response conserved DNA module |
| CAAT-box | -69(+), -99(-), -240(-), -246(-), -523(-), -537(-), -543(+), -546(-), -613(-), -691(+),-723(-), -776(+),-807(-),-819(+) | GGGTCAATCT | The promoter and the enhancer act together as cis-acting elements |
| CGTCA/TGACG-motif | -310(-)/(+) | CGTCA | Participate in MeJA response |
| ERE | -286(-) | ATTTTAAA | Ethylene response element |
| LTR | -323(+) | CCGAAA | Low temperature response element |
| **bHLH** | **-691(-),-723(-),-758(+),-806(+)** | **CANNTG** | **related to the regulation of the bHLH transcription factor family** |
| P-box | -667(-) | CCTTTTG | Gibberellin response element |
| TGA-box | -310(+) | TGACGTAA | Participate in MeJA response |
| TGA-element | -409(+) | AACGAC | Auxin response element |
| Box_1 | -219(-) | CGTGG | Optical response element |
| Box_4 | -321(+),-498(-),751(-),-788(+),-837(+) | CTCC | Conserved DNA modules involved in light response |

**Table S3.** Calibration curves and correlation coefﬁcients of CGA, CA, rutin, and luteolin

| Phenolic acids | Calibration curve^a^ | r^2^ |
| --- | --- | --- |
| Chlorogenic acid | Y = 22.19X - 42.11 | 0.9999 |
| caffeic acid | Y = 72.83X - 89.20 | 0.9999 |
| rutin | Y=994.59X + 0.3792 | R2=0.9997 |
| luteolin | Y=1907.1X + 2.596 | R2=0.9997 |

Y and X are the peak area and concentration of the Phenolic acids, respectively. Data are means ± standard deviations (SD) (n=3).
